# Response to: “Criteria for determining if a treatment for pain works”

**DOI:** 10.1016/j.inpm.2022.100154

**Published:** 2022-11-05

**Authors:** Ron D. Hays, John Devin Peipert

**Affiliations:** University of California Los Angeles, USA; Northwestern University Feinberg School of Medicine, USA

Dear Editor

We read the article by Bogduk [1] on “Criteria for determining if a treatment for pain works” with great interest because of the importance of the topic. Unfortunately, the 7-page commentary was riddled with problems that will confuse readers rather than enlighten them.

Table 2 contains the same information as Table 1 except it adds the mean values for the before and after scores and the significance level for a two-sample *t*-test. Both tables have a typographic error for the 8th case in the “After” score column (“9.9” should be 7.9). Because the hypothetical study is about data for 10 individuals before and after a treatment, the paired *t*-test reported in Table 1 is correct and the two-sample *t*-test reported in Table 2 is wrong as is Bogduk's statement that “the patients as a group have not changed … state after treatment is indistinguishable statistically from their state before treatment” (p. 3). Similarly, a paired *t*-test and Wilcoxon signed rank test should have been used instead of a two-sample t-rest and Mann-Whitney test in the second example (Figs. 1–2) and the third example (Figure 3).

The author provided a list of “minimal clinical important changes” for different outcome measures in pain treatment (Table 3) without noting that interpreting the magnitude of these changes can only be understood considering the scale of measurement and SD of the measure.

In a third example, the author claims that “despite being statistically significant, a mean improvement by 1.6 does not constitute evidence that the treatment has worked” (p. 4). The SD of change was 0.6 meaning that the effect size was 2.7! If this isn't a large enough difference to constitute a minimally important change, then what is?

One of the rows of Table 3 of the article indicated a value of 8 as the minimal clinically important change for the PROMIS physical function scale based on a study by [2]. Because this measure is scored on a T-score metric (mean of 50 and SD of 10 in the U.S. general population), 8 is 0.8 SD [3]. noted that this estimate is implausibly large because the methods used to estimate it were flawed. In contrast, the minimum detectable change (MDC), or coefficient of repeatability, refers to the minimum amount of change required for statistically significant individual change. The size of the MDC is directly related to the standard error of measurement. The MDC was used incorrectly as an estimate of the minimally important group change (the MCID) by [2]. Estimates of the MCID from the retrospective rating of change anchor item were based on all those that changed (much worse, worse, slightly worse, slightly improved, improved, much improved) rather than restricting the estimate to people that have changed by a minimal but important amount (slightly worse or slightly improved).

It is worth noting, that evaluation of group change is different than individual change. The amount of change required for significant individual change is typically much larger than what is needed for statistically significant group mean change because the denominator to assess individual change reliability exceeds the standard error of the mean. That is, when the group sample size is 6 or larger and the reliability of the measure is 0.90 or less, the amount of group mean change needed to be statistically significant will be less than the amount needed for statistically significant individual change [4].

## Funding

Dr. Hays was supported in part by the analysis core of the 10.13039/100007185UCLA Resource Centers for Minority Aging Research Center for Health Improvement of Minority Elderly RCMAR/CHIME) under 10.13039/100000002NIH/10.13039/100000049NIA Grant P30-AG021684.

## Declaration of competing interest

The authors declare that they have no known competing financial interests or personal relationships that could have appeared to influence the work reported in this paper.
